# *Malaria eradication in Mexico*: *Some historico-parasitological views oncold war, deadly fevers *by Marcos Cueto, Ph.D

**DOI:** 10.1186/1747-5341-3-15

**Published:** 2008-06-02

**Authors:** Filiberto Malagón

**Affiliations:** 1 Laboratorío de Malaríología, Facultad de Medicina, Universidad Nacional Autónoma de México, Ciudad Universitaria Coyoacán, 04510, Mexico DF, México

## Abstract

This review of Professor Marcos Cueto's *Cold War Deadly Fevers: Malaria Eradication in Mexico, 1955–1975 *discusses some of the historical, sociological, political and parasitological topics included in Dr. Cueto's superbly well-informed volume. The reviewer, a parasitologist, follows the trail illuminated by Dr. Cueto through the foundations of the malaria eradication campaign; the release in Mexico of the first postage stamp in the world dedicated to malaria control; epidemiological facts on malarial morbidity and mortality in Mexico when the campaign began; the emergence of problem areas that impeded eradication; considerations on mosquitoes and malaria transmission in Mexico; the role of business and society in malaria eradication; the results of the campaign; the relationship between malaria and poverty; and the parasitological lessons to be learned from the history of malaria eradication campaigns. Dr. Cueto's excellent and well-informed exploration of malaria – not merely as a disease but as a social, economic and human problem – makes this book required reading.

## The book

*Cold War, Deadly Fevers: Malaria Eradication in Mexico, 1955–1975 *is a historical account and analysis of the World Health Organization's global malaria eradication program as it unfolded in Mexico. The author, Marcos Cueto, a historian and a professor in the department of sociomedical sciences at the School of Public Health at the Universidad Peruana Cayetano Heredia in Lima, reveals with x-ray precision the structure underlying the global effort to conquer malaria, launched in 1955; like a portraitist, he also shows the outer peculiarities this structure assumed in specific locations.

Dr. Cueto vividly describes the experiences, policies, and scientific beliefs on which the global malaria eradication program was built. According to him, the United States supported the program to increase its presence in Latin America and other regions afflicted by malaria – less as a public service than as a strategic blow to creeping communism, and even as a business opportunity.

Dr. Cueto's superbly well-informed exploration of malaria not only as a disease but as a social, economic, and human problem makes his book required reading. The only extant comparable work is *El Paludismo en America Latina *[1], published from a thesis written by Dr. Saul Franco-Agudelo to obtain his master's degree in social medicine at the Universidad Metropolitana of Mexico City.

As a Mexican parasitologist dedicated for many years now to the study of malaria parasites, I found Dr. Cueto's excellent book especially exciting. Think of these comments as a trailer for the book, which gives its reader a front-row seat for the unfolding of malaria eradication in Mexico.

## Foundations of the malaria eradication program

The history of malaria eradication in Mexico and the world is the story of dichloro-diphenyl-trichloroethane (DDT), a compound first synthesized in 1874. It had no known application but was resynthesized in 1939, at which point it was recognized as an effective insecticide. DDT was used during the Second World War to control lice, fleas, and mosquito-transmitted diseases, and it was soon established as the most useful tool for controlling insect vectors of public health importance. DDT campaigns successfully controlled malaria in Europe. Its effectiveness encouraged the notion that malaria could be entirely eliminated across the globe. When the war ended, DDT was commercialized and made part of malaria control programs in various tropical countries, including Mexico.

The WHO Eighth World Health Assembly, held in Mexico City in 1955, approved the global malaria eradication campaign. This program was to be carried out in Europe, North Africa, the Middle East, some Asian countries, and Latin America. Sub-Saharan Africa was deemed "not ready" [2] and excluded from the supposedly "global" initiative, which therefore ignored the territories that produced 90% of the malaria cases and deaths in the world at that time. This statistic bears repetition: the countries that produced just 10% of earth's malaria infections were the only ones with which the global malaria eradication program concerned itself. The program succeeded in driving malaria out of southern Europe, North Africa, the Middle East, and some islands in the Atlantic Ocean. India and Sri Lanka almost eliminated malaria, but then it returned dramatically.

At the World Health Assembly in 1955, Latin American governments agreed to finance the campaign for five years, the amount of time WHO had deemed sufficient for eradication. Altruistic US funds (administered through UNICEF and what was then called the Pan American Sanitary Bureau, today the Pan American Health Organization) were the fuel that got the campaign going in Latin America.

But in 1960 not one of the countries of continental Latin America was free of malaria. The campaign was extended. By 1966, it looked as if Mexico might achieve eradication, but by 1970 that promise was shown to be false. Even after WHO dissolved its global efforts, Mexico's campaign went on until 1986, by which time malaria had once more spread throughout its original area of incidence and malaria morbidity had reached an alarming 140,000 cases. But eventually all countries gave up on eradication and implemented programs to control malaria, using the instruments and techniques left over from the era of eradication. Today, Mexico officially reports from one to three thousand cases annually.

DDT's efficacy during and after the Second World War made global malaria eradication seem feasible; this conviction was further supported by the following experience-based assumptions:

1. Malaria transmission occurs at night inside human dwellings, where anopheles mosquitoes bite people to feast on their blood.

2. Anopheles mosquitoes remain within human dwellings after sucking blood and rest on their interior walls.

3. Malaria parasites have a finite lifespan in human hosts, if treatment is not administered and infection is left to run its natural course. *Plasmodium falciparum *die in six months to one year; *Plasmodium vivax *survive inside human beings for one to one and a half years (and sometimes more); and humans can host *Plasmodium malariae *for 10 to 40 years or until they themselves die. Finally there is *Plasmodium ovale*, which is no concern of ours because this species has not been demonstrated to exist in the Americas.

4. The application of persistently-acting insecticides to a house's interior walls is sufficient to interrupt malaria transmission.

5. If transmission were interrupted for just three years (later upped to five), malaria would be eradicated.

6. The spraying must be done as quickly as possible, before mosquitoes develop resistance to insecticides.

Theoretically, what could be expected if these assumptions prove to be true?

When insecticide spraying began in these communities, they were home to adult mosquitoes (in the air) and to larval mosquitoes (in nearby bodies of water). The plan was to spray the walls of every single house. Mosquitoes in search of a place to rest after sucking human blood tend to land on the walls of dwellings, and all mosquitoes that landed on sprayed walls would die. Female mosquitoes – the ones that suck blood, and therefore the ones that transmit malaria – do not feed every day; they take a meal every third or fourth day in order to lay eggs. They do not, of course, all choose to feed on the same night, but it could be reasonably supposed that all adult female mosquitoes would feed on blood at least once over the course of a week. Thus, within a week of commencement of spraying, all mosquitoes that were adults on day one would have fed and then died after coming into contact with a sprayed surface.

Once the adult mosquitoes were dead, however, the biting would not have ended for good, since immature mosquitoes, unaffected by the spraying, would eventually reach the adult stage and begin their own quest for blood. Each egg present on water bodies at commencement of spraying would develop through five larval stages and one pupa, from which the adult hatches. This process takes about 20 to 30 days at tropical temperatures, so within 30 days a new anopheles mosquito population would emerge. (There should be no eggs laid after commencement of spraying, since spraying would terminate extant adult mosquitoes before they could lay eggs.) This new population would die as its forbears did, on the sprayed walls of houses they had entered in search of a meal of human blood.

If the assumption was correct, then about 30 days after the first DDT spraying the mosquito population would be eliminated and transmission would be interrupted, with no females at any stage of development left to transmit malaria. Sprayed insecticide would remain potent for five months even without further spraying, which would take care of any mosquitoes that had escaped death. Once female mosquitoes were eliminated, the only source of malaria parasites would be the blood of people who were already infected when the eradication campaign began.

Insecticide spraying coupled with antimalarial treatment should have eradicated malaria in one or two months. The WHO campaign manual for the eradication of malaria did not call for treatment, however; instead it recommended five full years of spraying, during which time all parasites would die naturally inside their human hosts.

Why wasn't treatment administered to people with malaria as their houses were sprayed, when this combination would, theoretically, have eliminated a community's parasite population in just thirty days, setting the stage for eradication maintenance? According to Dr. Cueto, Mexico and other countries were familiar with DDT since 1945 on, first sprayed experimentally and later routinely used in malaria control campaigns and, that is why there was a big push within WHO to start the campaign before the mosquitoes developed resistance to the insecticides: once they developed resistance, it was thought, eradication would be impossible. Why wasn't treatment administered along with the spraying? Because WHO thought spraying was enough, and more efficient than treatment? Because spraying without treatment would allow malaria to linger and the insecticide manufacturers to do more business? We cannot say.

In any event, many of the eradication campaign's initial assumptions turned out to be wholly or partially incorrect, and Dr. Cueto describes a host of other unforeseen troubles that plagued its efforts. What is clear today is that the failure to provide treatment to people suffering from malaria was inhumane.

## The mosquito postage stamp

**Figure 1:**
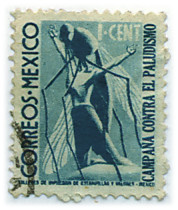
**El timbre del mosquito**. Mexican postage stamp dedicated to malaria control.

Dr. Cueto recreates Mexico's atmosphere in the years prior to the eradication campaign. As part of this atmosphere, in 1938 the Mexican government had decided to make malaria a national priority (at least the second time it had done so), establishing the Comision de Saneamiento Antimalarico and funding it with 15% of the Ministry of Health's total budget [3]. But these good intentions were never realized, and the Commission actually operated with much more limited funding. Everyone wanted to solve the malaria problem, but there wasn't enough money to do so. Out of this abundance of enthusiasm and dearth of financing came a postage stamp dedicated to malaria control; its sale would raise funds for Commission operations [4]. The stamp known as the *timbre del mosquito *(the mosquito postage stamp) came out in 1939; it was the first postage stamp in the world dedicated to malaria control [5]. As seen in figure 1, the stamp was worth 1 Mexican cent. Printed in Prussian blue, it shows a kneeling, bare-chested man raising his arms and curling his fingers, as a gigantic mosquito bites him. Man at the mercy of mosquito: an accurate description of how people felt about malaria at that time (see Figure 1).

## Establishing a base line

In order to measure the progress of the malaria eradication campaign, some figures on malaria morbidity and mortality in Mexico had to be established before operations commenced. Different estimates existed, but the most widely accepted at the time was a figure of 2 to 2.5 million cases of malaria per year with an annual mortality of 25,000 persons [6,7].

These numbers, however, do not hold up to analysis. It is evident that those 2.5 million supposed malaria cases were not diagnosed on parasitological or clinical grounds. Malaria diagnoses rely on the clinical skill of physicians, and at that time there were only around 16,000 doctors in all of Mexico, most of whom practiced in major cities. Most malaria cases, on the other hand, occurred in small towns or villages in rural tropical areas, where there were few if any physicians. Diagnoses were not commonly based on the evidence of parasites in the patients' blood. So who was diagnosing malaria, if we know that physicians were not where the malaria was? These must have been postmortem diagnoses, made with neither autopsies nor licensed physicians. It was the civil servants in small towns who would record deaths; they would ask relatives for the cause of death or would invent one if necessary. Malaria had about 22 different popular names throughout Mexico: yellows, fevers, fever between body and flesh, spleen fever, colds and fever, intermittent fever, chauiste (a Nahuatl word, originally referring an ill plant that develops black spots in its leaves, perhaps from a fungal infection. Chauiste falls on humans in the form of ill tidings or disease), etc. [8]. When these records were submitted, the names assigned to the causes of death had to be translated by the Ministry of Health, where, in each case, an expert would decide what the popular name chosen by the local civil servant meant in medical terms and thus produce the official final diagnosis. With this diagnostic technology, 25,000 annual deaths for malaria were recorded previous to 1955. Morbidity was projected from the number of deaths, based on the assumption that one person would die out of every hundred people infected: 2.5 million cases.

This reckoning accounted only for the most deadly *Plasmodium *species, *P. falciparum*. But according to the recognized pathogenicity of the four *Plasmodium *species that thrive in humans, *P. malariae*, the most benign, almost never kills its host (unless he or she develops a complication); and *P. vivax *and *P. ovale*, though they can produce very severe clinical states, are also considered benign, for they usually do not kill their human host in the absence of another disease (such as pneumonia). The only species that kills its host singlehandedly, as it were, is *P. falciparum*. In 1958 when blood parasite estimations were done, *P. falciparum *accounted for 14%, *P. vivax *for 85% and *P. malariae *for 1% (rounded figures) of infected people [9].

If there were, as estimated, 2.5 million true malaria cases, the species breakdown should have been: *P. falciparum *350,000; *P. vivax *2,125,000; and *P. malariae *25,000. Again using an estimated one death per 100 cases, the total number of deaths should have been 3,500, which is quite different from 25,000.

If there were really 2.5 million cases occurring annually among 15 million inhabitants (people living in areas affected by malaria), then one inhabitant in six was infected before the campaign. After one year of the campaign, one inhabitant in 150 was infected (calculated from [9]); and five years into the campaign, only 25% of the original malarious area (more than one million square kilometers) was plagued by residual malaria [10].

We'll never know for sure how many Mexicans were infected with malaria before the campaign. Therefore we can never know how much of the decline in the official number of malaria-infected people should be attributed to DDT spraying and how much to initial overestimation of the morbidity.

## The problem areas

Dr. Cueto demonstrates that intervention plays out differently in different geographical regions; the methods that succeed in one place will not necessarily work in others, even within the same country. Soon after the campaign began it became clear that the same eradication scheme did not produce the same results everywhere. After five years of insecticide coverage (1960), malaria had almost been eliminated from the Gulf coast; along the Pacific cost from Sinaloa to Chiapas, however, malaria was still being transmitted in many areas [11]. One year later, a new phrase was coined in Mexican eradication jargon, "problem areas," which referred to those regions where malaria transmission persisted [12]. Later the term "problem areas" came into use internationally.

Dr. Cueto points out that Mexico's problem areas were also its poorest areas. They tended to have low but constant malaria transmission, no matter what eradication techniques were used (malaria treatment, larvicides, sanitary engineering of mosquitoes' favored breeding places, and insecticide fumigation of mosquitoes' outdoor haunts); and most of them were villages built on river banks or otherwise very close to rivers. Furthermore, the expectation had been that the program, properly implemented, would eradicate malaria in five years. When five years passed and malaria had not been eradicated, demoralized eradication workers began to do their work sloppily. Thus, from the problem areas malaria could spread once more throughout its old area of incidence, throughout areas where it had supposedly been eradicated.

## Mosquitoes and malaria transmission

Malaria parasites cycle from man to mosquito and from mosquito to man without ever coming into direct contact with the environment. A campaign aiming to stop malaria transmission must find a way to measure the state of transmission at any given moment in the communities being sprayed. An indirect way is to measure the parasites circulating among the community's people; the direct way is to measure the parasites circulating in the stomachs and salivary glands of the Anopheles mosquito population. Estimations of the number of infected people in a community are usually based on the demonstration of malaria parasites in the blood of subjects who are experiencing febrile episodes or who have experienced such episodes in the previous 30 days. But there are people who have malaria but do not exhibit symptoms, and though they are spreading their parasites to the mosquitoes that bite them, they are not accounted for in the estimations from febrile subjects. The only accurate way to quantify the state of transmission is to measure malaria infection in the mosquito population.

There are very few entomological studies of mosquitoes' infectivity in Mexico's malarious areas. The Rockefeller Foundation Archives dating back to even prior to the eradication campaign show how some Mexican government and Rockefeller Foundation officials felt about the campaign's agendas regarding entomology and malaria. Wilbur Downs, director of Rockefeller's malaria control program in Mexico from 1946 – 1952, complained of the Mexican government's tendency to undertake DDT campaigns without first investigating the incidence of malaria and of the principal anopheline vectors; on the other hand, Fred Soper, perhaps the principal promoter of the eradication campaign, believed that long-term, detailed entomological or malaria studies would be necessary only if DDT failed. Later a Mexican public health officer opined that a DDT based malaria control program did not require malariologists, engineers, or entomologists at all [13].

Records on mosquito entomological work in Mexico exist since 1885, but as far as we know, (since Luis Vargas, the entomologist of the eradication campaign did not mention studies done on *Anopheles *mosquitoes infectivity in his historical data on Entomology of Malaria in Mexico [14]), it was not until 1955, when the National Comission for Malaria Eradication was created, that for the first time systematic entomological studies on mosquito vectors were carried out. Personal communications from the campaign's entomologists reveal that malaria-infected mosquitoes were uncommon but do not say anything about the malaria transmission dynamics observed; and so it seems that infected and infective mosquitoes were casually studied but not in ways that ever became known outside the campaign organization. This fact was exposed in 1970 when the vocal executive of the malaria eradication campaign in Mexico proposed another Six Year Plan to complete eradication [7]. The plan described explicitly everything that needed to happen. Entomological studies were required: anopheline density, antropophilia (man as food source), endophilia (food taken inside man's house), susceptibility to insecticides, longevity, and resistance to insecticides. But there was no intention to determine sporozoite and oocysts rates in the vector mosquitoes-that is, there was no intention to study the infective forms. We know this was not an involuntary omission since such studies had not been done in quite a long time, and, if done, no one had published the results. We might wonder how many other campaigns in Latin America were skipping this important step.

Interesting malaria epidemiological work had been done in Mexico in 1935 by Professor Bustamante [15] in Xochimilco and adjacent villages. He employed classic malariological procedures, including an entomological study in which he established that *Anopheles occidentalis var aztecus *(later referred as *A. aztecus*) was the chief malaria vector (in the Valley of Mexico) of *P. vivax*, the prevalent parasite species. This study observed infected (oocysts in stomach) and infective (sporozoites in salivary glands) adult mosquitoes by dissecting 200 mosquito stomachs and 50 salivary glands. Data from these dissections were not included in the paper. The author defended their omission, arguing that these observations were not publishable because the technicians who had performed the dissections were in the process of being trained. One wonders now if any infected mosquitoes were found. If there were no infected mosquitoes but there was malaria, how could it be said that mosquitoes were the only possible transmitters of the disease? Could there be other means of transmission, ones that might explain the existence of problem areas in which transmission persisted even when the amount of DDT sprayed was doubled or tripled and other interventions were employed? What about the possibility of malaria transmission by mouth [16]?

## Society and business in malaria eradication

Once upon a time two rival political and social orders vied for global political and social domination. One, which came to existence early in the twentieth century, declared that it wanted to build a classless society in which no man would be exploited by any other. This meant that private property would eventually have to be abolished. Instead of individual owners, all means of industrial production would have a single faceless owner, the government, which would distribute profits equally among all members of society. The result, it was said, would be a truly egalitarian society with no thieves, no poverty, and no unemployment. This world was represented by just one country, but this country had its satellites, and together they were called the communist world or the second world.

The other world was called the first world, since it had been around since the Renaissance. In this world, the family and the individual were the basic units of society, and individual freedom was of paramount importance. Every member of the society was free to own anything he could afford and to be the boss of other people, thereby profiting from their work. Men were limited only by their capacities to accumulate. It was a world of competition, and wealth in this world was unevenly distributed, for each person took whatever he could earn or steal. There were three classes: the rich, the middle class, and the poor. This world emerged at more or less the same time in several European countries, which went on to colonize and gobble up many regions, countries, and cultures that until then had had their own ways and social structures. This was the capitalist world, and it exploited the poor nations of the world even as capitalist bosses exploited the poor workers within their own countries.

After the Second World War, the United States (the foremost representative of the first world) and the Soviet Union (the leader of the second world) struggled for control of the earth's peoples. The goal of the first world in this Cold War was to preserve its hegemony by encouraging the development of capitalist countries and fighting communism wherever it was to be found. The second world's goal was to convert all poor countries to communism and thereby to bring an end to exploitation and poverty. This is the atmosphere in which the global malaria eradication program, a first world initiative, was launched. Others have portrayed the vicissitudes of the rise and fall of the malaria eradication campaign as a struggle of strong personalities, idiosyncratic choices, historic ironies, and unintended consequences [17]. By 2007, Dr. Cueto's characterization of this campaign in the light of USA hegemony, an interpretation shared by other authors, as is signaled by Stapleton [13], has substantial and illuminating validity.

As it turned out, some citizens of the communist world were more equal than others. Although some small pockets of communism remained, most communist societies returned to capitalism and individual liberties. As these societies were crumbling back into capitalism, the malaria eradication program was dying: WHO canceled it in the 1980s. Even when the worldwide malaria eradication program died, national malaria control programs continued their work and still work today.

Communism evaporated, the cold war came to an end, and the previously balanced world was left with a single hyperpower, the United States. The United States continued to push market freedom, tearing down frontiers throughout the world – not for individuals but for goods and capital. Monopolies came into being as big corporations engulfed smaller ones, creating tremendous hoards of capital and power. Corporations dominated national governments and thus the world in all its economic, cultural, political and social expressions. Even in the former Soviet Union, consolidation and power-grabbing were the name of the game as oligarchs took control of what had been the state's. Like the old communist superbosses, giant corporations controlled the means of production; under capitalism, however, these bosses gave no thought to sharing profit with workers. These corporations made sure their advocates were well placed within governments and international organizations in order to influence policy decisions. Now corporations decided what citizens should read, hear, see, and taste, how they should be educated and what social principles would govern them. This is the story of the world we live in now, and its moral is that given limitless liberty, a bewildered or uncomprehending people may choose to allow others to make their choices for them without asking for their consent. Where then is the land of liberty, if, at a personal or international community level, the liberty of one is abolished for the sake of the liberty of another?

But what was the role of malaria eradication in this story? The malaria eradication campaign left a door open for The United States to bring the third world of Latin America into its fold during the Cold War, losing just one country, Cuba. To eradication campaign operations the United States contributed not cash but equipment and materials, so the donated money was almost entirely returned to the USA or never left (because donated money was largely used in the USA to pay for malaria eradication technology which was then exported). In most countries the amount donated to support the campaigns did not cover all the expenses. According to Dr. Cueto, Mexico received disproportionately large support among Latin American countries, and yet from 1956 to 1964 the Mexican government had to pay 75% of all the campaign expenses [3]. Mexico and other Latin American countries had to make up the budget shortfall in order to implement the program by buying American technology. After the first five years of operation, American donations dwindled, and some years later they wholly disappeared. At that point all countries had to pay for 100% of their own programs even as they continued to purchase malaria eradication technology from the United States. Countries engaged in eradication programs chose to continue in the same path for many years rather than innovate; in fact, all those countries fight malaria today with the same tools, barely modified. Healthier local populations in malarious countries benefit American companies operating there, so whatever help the United States did offer could not be considered selfless. The United States was selling a package of malaria eradication technology and instructions for using it at a cut rate – essentially buy one, get one free! – to create clients. The truth is that the US was, with only minimal investment up front, opening a market for its products that is still open today.

Buying and selling are facts of life in our modern world. The seller gives the purchaser a good, and the purchaser gives the seller some money. But if the good does not satisfy the client, the seller should refund his money. And in the case at hand, Mexico and Latin America were sold a most unsatisfactory bill of goods.

The malaria eradication campaign did not eradicate malaria, not in the time promised, and in fact not at all. In the first world even health care and education are thought of as consumer goods; so who's to say that malarious countries do not have the right to demand a refund? Because the transaction was disguised as humanitarian help, the cheated states will never see a refund.

The future of malaria control intervention is population-wide vaccination in malarious countries. We may also see on the market satellite services that predict malaria epidemics and alert susceptible countries. None of this is bad, but malarious countries should remember to keep their receipts, even if it seems as if they're getting a good deal.

## The results

More than half a century has passed since the malaria eradication campaign began in Mexico. The campaign and the control program that followed it reduced malaria mortality to zero; after ups and downs in morbidity, there were 3819 cases in 2003, Dr. Cueto notes. The official morbidity figures today are similar, and they are also quite similar to the morbidity figures obtained at the high point of the eradication campaign (1958–1959). Then everyone believed that malaria was coming to an end. Now it once again appears that eradication is succeeding and that the end of malaria is at hand. If the currently accepted morbidity rates are accurate, it would be reasonable to expect malaria to be eradicated within a short time.

If eradication does occur, we will have to ask how much of this success came as the result of DDT spraying and other interventions, and how much was due to unrelated economic and social changes. We might ask ourselves what the malaria situation and socioeconomic picture would look like today if the past fifty-one years had been spent trying to eradicate not malaria but poverty, a campaign that would have included environmental sanitation, medical attention, employment programs, social organization, and education.

## Malaria and poverty

Poverty begets disease and disease begets poverty. As do other diseases, malaria spins a vicious circle in which poor people with malaria become poorer and therefore more vulnerable to malaria. Malaria prospers most where human societies prosper least [18].

What does poverty have to do with malaria, if malaria is transmitted by mosquitoes? Mosquitoes do not discriminate between rich and poor; they'll bite anyone with blood coursing through his veins. But the poor have poor housing and thus are more exposed to mosquito bites. Impoverished communities frequently become environments in which mosquitoes (not to mention pathogenic bacteria, parasites and fungus) thrive. Caught in cycles of unemployment and low-paying jobs, poor people generally have very few opportunities to improve their conditions. Frequently their nutrition is very poor, leaving them vulnerable to malaria and other diseases.

The Mexican experience with malaria eradication demonstrated the connection between malaria and poverty. Even the United States provides examples of this connection. According to Professor Faust [19] the malaria east endemic area was divided into two regions: one in the Northeast, where winters are cold and summers are short and periods of transmission are therefore limited; the parasite there is *P. vivax*. The other region is the hyperendemic south, where a subtropical climate allows for year-round transmission of malaria by both *P. vivax *and *P. falciparum*. In the north, agricultural improvement and desiccation of mosquitoes' breeding places rendered malaria sporadic, while in the south, where the population was poorer, it is hard to say which interventions helped decrease malaria infections. It was probably the combined effect of a number of programs applied to boost the southern economy, social services, and public health services.

## Some parasitological lessons from history of the malaria eradication campaigns: An opinion

Malaria is not only the result of a host-parasite relationship. Environmental, economic, and social factors also play important roles. Best results of insecticide spraying are obtained in a relatively brief time if coverage is total, but best results can only be obtained where socio-economic indicators are better. This is because antimalarial intervention alone can lead to a decrease in morbidity, but it will only be temporary; morbidity will climb back up when intervention is interrupted or diminished. Antimalarial intervention must coincide with environmental, economic and social improvements, or a chronic intervention over the course of many years will be necessary simply to preserve unstable initial benefits.

When chronic antimalarial intervention alone is applied, it is possible that malaria will disappear eventually, after many years. This is, however, not only the result of the intervention, but also of the unrelated, naturally occurring social evolution of communities, which at a certain point removes the environmental, economic, and social elements that facilitate disease. The disease disappears when antimalarial interventions are combined with permanent environmental, social and economic changes in the malarious communities.

Malaria control technologies themselves have barely evolved. DDT is being used again in Africa, despite its drawbacks.

If we want to vanquish malaria, our proper foe is not the mosquito; it is poverty. Universal human rights to health, food, and education are just words on paper and in speeches today. We need to make these wordy dreams into facts, for today the rights we like to think of as universal are simply out of reach for the world's poor.

## About the Author

Professor Malagon, M.D. (from the Faculty of Medicine of the Universidad Nacional Autonoma de Mexico), and Msc (from the London School of Hygiene and Tropical Medicine), is dedicated to teaching medical parasitology and malaria to pre- and post-graduate students and to research in malaria. He has been appointed President of the Mexican Society of Parasitology, and President of the Mexican Association of Microbiology and Parasitology Teachers in Faculties of Medicine. Head of the Malariology Laboratory at the Faculty of Medicine in the University of México, he is Profesor afiliado of the Faculty of Medical Sciences at the San Carlos University in Guatemala.

## Competing interests

The author declares that they have no competing interests.

## Authors' contributions

Professor M is responsible for the entire manuscript.

## References

[B1] Franco-Agudelo S (1990). El paludismo en América Latina.

[B2] Martin S, Alilio IB, Bygbjerg C, Breman JG (2004). Are multilateral malaria research and control programs the most successful? Lessons from the past 100 years in Africa. Am J Trop Med Hyg.

[B3] Fajardo-Ortiz G, Carrillo AM, Neri-Vela R (2002). Perspectiva Histórica de Atención a la Salud en Mexico, 1902–2002.

[B4] Cabrera-Palma J (1961). La lucha antimalárica y la filatelia en Mexico. Boletín CNEP.

[B5] Doby JM, Canning EU (1981). Paludisme et timbres-poste. Parasitological Topics, Society of perotozoologists Special publication No1.

[B6] Pesqueira ME (1957). Programa de erradicación del paludismo en Mexico. Boletín de la Oficina Sanitaria Panamericana.

[B7] Suarez-Torres G (1970). El programa de Erradicación de Paludismo. Plan de seis años. Salud Publica de Mexico.

[B8] Vargas L (1963). Realizaciones del programa de erradicación. Salud Publica de Mexico.

[B9] Marquez-Escobedo MB (1960). Estado actual de la erradicación del paludismo en Mexico. Boletín de la Oficina Sanitaria Panamericana.

[B10] Gomez-Mendoza I (1963). Perspectiva de la erradicación del paludismo en Mexico. Salud Publica de Mexico.

[B11] Alvarez-Amezquita J (1962). Estado actual de la campaña de erradicación del paludismo en Mexico. Salud Publica de Mexico.

[B12] Roman y, Carrillo G, Romero-Alvarez H, Gomez-Mendoza I (1965). Epidemiologia del paludismo residual en Mexico. Salud Publica de Mexico.

[B13] Stapleton DH (2004). Lessons of history? Anti-malaria strategies of the international health board and The Rockefeller Foundation from the 1920s to the era of DDT. Public Health Reports.

[B14] Varagas L (1970). La Entomologia de la malaria en Mexico. Prensa Medica Mexicana.

[B15] Bustamante M (1939). Epidemiología del Paludismo en el sur del Valle de Mexico. Zona de Xochimilco. Gaceta Médica de Mexico.

[B16] Malagon F, Tapia JL, Vazquez J, Robert L, del Toro F, Koning LO (2007). The oral transmission of malaria. Progress in malaria research.

[B17] Gladwell M (2002). Fred Soper and the Global Malaria Eradication Programme. J Public Health Policy.

[B18] Sachs J, Malaney P (2002). The economic and social burden of malaria. Nature.

[B19] Faust EC (1950). Un siglo de malaria en los Estados Unidos. Gaceta Médica de Mexico.

